# Biomarkers Associated with Death After Initiating Treatment for Tuberculosis and HIV in Patients with Very Low CD4 Cells

**DOI:** 10.20411/pai.v3i1.235

**Published:** 2018-04-26

**Authors:** Fred R. Sattler, Daniel Chelliah, Xingye Wu, Alejandro Sanchez, Michelle A. Kendall, Evelyn Hogg, David Lagat, Umesh Lalloo, Valdilea Veloso, Diane V. Havlir, Alan Landay

**Affiliations:** 1 Keck School of Medicine of the University of Southern California, Los Angeles, California; 2 Harvard School of Public Health, Boston, Massachusetts; 3 Social & Scientific Systems Inc., Silver Springs, Maryland; 4 Moi University Clinical Research Center, Eldoret, Kenya; 5 Enhancing Care Foundation, Durban University of Technology, Durban, South Africa; 6 Evandro Chagas National Institute of Infectious Diseases, Oswaldo Cruz Foundation, Rio de Janeiro, Brazil; 7 University of California San Francisco, San Francisco, California; 8 Rush Presbyterian Medical Center, Chicago, Illinois

**Keywords:** *Mycobacterium tuberculosis*, Human Immunodeficiency Virus, Timing of antiretroviral therapy, Predictors of mortality, Nutrition biomarkers, Innate immunity, Adaptive immunity

## Abstract

**Background:**

The risk of short-term death for treatment naive patients dually infected with *Myco-bacterium tuberculosis* and HIV may be reduced by early anti-retroviral therapy. Of those dying, mechanisms responsible for fatal outcomes are unclear. We hypothesized that greater malnutrition and/or inflammation when initiating treatment are associated with an increased risk for death.

**Methods:**

We utilized a retrospective case-cohort design among participants of the ACTG A5221 study who had baseline CD4 < 50 cells/mm^3^. The case-cohort sample consisted of 51 randomly selected participants, whose stored plasma was tested for C-reactive protein, cytokines, chemokines, and nutritional markers. Cox proportional hazards models were used to assess the association of nutritional, inflammatory, and immunomodulatory markers for survival.

**Results:**

The case-cohort sample was similar to the 282 participants within the parent cohort with CD4 <50 cells/mm^3^. In the case cohort, 7 (14%) had BMI < 16.5 (kg/m^2^) and 17 (33%) had BMI 16.5-18.5(kg/m^2^). Risk of death was increased per 1 IQR width higher of log_10_ transformed level of C-reactive protein (adjusted hazard ratio (aHR) = 3.42 [95% CI = 1.33-8.80], *P* = 0.011), inter-feron gamma (aHR = 2.46 [CI = 1.02-5.90], *P* = 0.044), MCP-3 (3.67 [CI = 1.08-12.42], *P* = 0.037), and with IL-15 (aHR = 2.75 [CI = 1.08-6.98], *P* = 0.033) and IL-17 (aHR = 3.99 [CI = -1.06-15.07], *P* = 0.041). BMI, albumin, hemoglobin, and leptin levels were not associated with risk of death.

**Conclusions:**

Unlike patients only infected with *M. tuberculosis* for whom malnutrition and low BMI increase the risk of death, this relationship was not evident in our dually infected patients. Risk of death was associated with significant increases in markers of global inflammation along with soluble biomarkers of innate and adaptive immunity.

## BACKGROUND

World-wide, tuberculosis remains the most common cause for hospitalization of HIV-infected patients with death occurring in 10%-30% of these patients [1, 2]. Recent studies indicate that death from tuberculosis can be reduced in HIV-infected treatment-naive patients by initiating anti-retroviral therapy (ART) earlier rather than later [3–5]. However, predictors of death in the ensuing months after ART is initiated have not been investigated in this co-infected population. In patients without HIV, malnutrition contributes to the incidence and severity of pulmonary tuberculosis [6–8]. Furthermore, wasting with very low BMI, malnutrition, and advancing age have been associated with a greater risk of death than for patients with better nutritional status, especially in developing countries [9–11].

Patients co-infected with tuberculosis and HIV often have worse nutritional status (inadequate intake of nutritional energy) with wasting (low BMI) and more cachexia (greater loss of lean tissue due to inflammation caused by HIV infection) than HIV-negative persons with tuberculosis [12–14]. Low CD4 cell counts are also associated with greater wasting and early mortality in persons co-infected with *M. tuberculosis* and HIV [15–17]. Wasting (low BMI) due to malnutrition and cachexia caused by inflammation in persons with HIV (without tuberculosis), which is often due to uncontrolled viral replication, are risk factors per se for death [6, 17–23]. Furthermore, poor nutritional status and low leptin (decreased energy intake) may suppress cellular immunity thereby increasing the risk for adverse outcomes. Regardless of the cause of wasting and cachexia, death has been extrapolated to body weight of 66% or body cell mass of 54% of pre-morbid levels in persons with HIV [24]. However, the exact mechanisms contributing to death in persons co-infected with *M. tuberculosis* and HIV have not been determined.

The conceptual underpinning for this investigation is that death in HIV-positive individuals acutely infected with *M. tuberculosis* and who are initiating treatment for the first time with combination ART is due in part to malnutrition from impaired energy intake. We also postulate that this co-infection induces an intense, self-perpetuating cytokine cascade beginning with the local production of TNFα and interferon gamma (IFNγ) in response to *M. tuberculosis* in the lung. These cytokines induce cellular signal transduction that promotes NFκβ nuclear translocation and transcription of gene products [25], ultimately resulting in the production and release of a broad array of pro-inflammatory cytokines into the systemic circulation, that also enhance HIV viral replication systemically and locally in the lung [26, 27].

HIV infection independently stimulates nuclear transcription through NFκβ, which upregulates both viral replication and translation of gene products to increase production and release pro-inflammatory mediators [18, 28]. In addition, high levels of viral replication are associated with greater wasting [19, 29] and are expected to impair immune recovery including pathogen specific immunity for *M. tuberculosis*. These 2 infections together are thus expected to cause auto-amplification of pro-inflammatory cytokine pathways, thereby resulting in more severe cachexia with loss of lean tissue mass and thus enhanced risk of death [20, 21], especially in the early period of treatment for tuberculosis [15].

In the AIDS Clinical Trials Group Study A5221 in which ART was begun early or deferred by approximately 1 month, there was an increased incidence of death plus new opportunistic infection in the ensuing year in participants with baseline CD4 counts < 50 cells/mm^3^ who delayed ART by 1 month. The *a priori* goal of this secondary objective of A5221 was to determine if baseline measures of 1) nutrition or 2) inflammation and immune activation could be related to death in the participants initiating therapy for HIV and tuberculosis who had low CD4 counts (< 50 cells/mm^3^) [3].

## METHODS

To address our first hypothesis that poor nutritional status contributed to death in the study cohort, BMI, albumin, hemoglobin, and leptin levels were compared in participants who died versus those who survived. For the second postulate that inflammation and/or immune activation contributed to death, levels of C-reactive protein (CRP), a number of pro-inflammatory cytokines, anti-inflammatory cytokines/ligands, chemokines, and measures of the innate and/or adaptive immune responses were compared in participants who died versus those who survived within the case-cohort sample. All participants provided informed consent prior to enrollment in the ACTG A5221 study.

### Study design

Our investigation used a retrospective case-cohort design: a random sample was drawn from the parent study and then all cases that were not selected in the random sample were added to make the full case-cohort sample. The case-cohort design combines the advantages of a prospective cohort study and the efficiency of a case-control design. It is most useful in analyzing time to failure in a cohort in which failure is rare. For the purposes of this investigation, a case (failure) was defined as death occurring in the 48 weeks after study enrollment.

### Study population

The parent cohort is based on the ACTG A5221 study, which was a randomized, open-label 48-week investigation comparing earlier versus later ART in persons with HIV-1 infection and suspected or documented pulmonary tuberculosis and with CD4 counts of < 250 cells/mm^3^. A total of 806 participants were eligible and enrolled in A5221. Of these, 282 participants had baseline CD4 (average CD4 count over screening and study entry visits) < 50 cells/mm^3^ [3].

**Figure 1.**
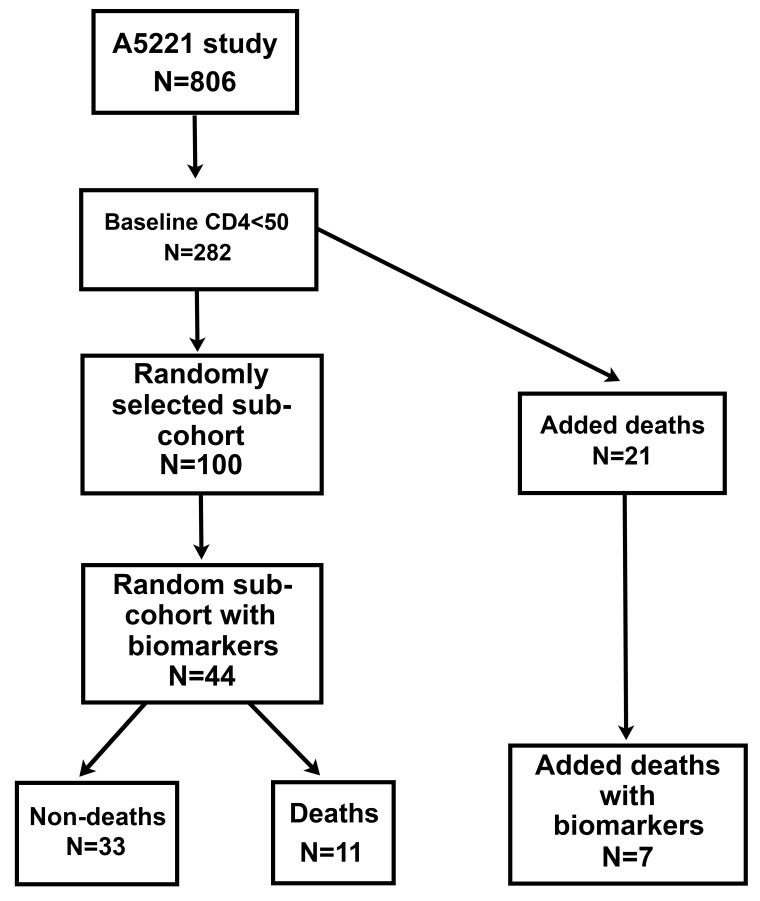
**Schema for Selection of the Case-Cohort.** A random cohort consisting of 100 participants was drawn from a subset of the 806 study participants who had CD4 < 50 cells/mm3 (n = 282). Plasma was only available for 44 participants in this subset, 11 of whom died. This cohort was enriched by selecting 21 additional cases from the 282 participants having CD4 < 50 cells/mm3 (not in the random cohort of 100) who also died, but only seven of these had plasma. The total case-cohort for the study therefore included 51 participants

For this investigation, a random cohort of 100 participants with baseline CD4 <50 cells/mm^3^ was drawn from the 282 parent-study cohort. Of these, 44 participants had sufficient baseline plasma samples stored for biomarker evaluation; 11 of the 44 died during the study. An additional 21 cases of death from the parent cohort with CD4 < 50 cells/mm^3^ were also included, but only 7 had sufficient baseline plasma samples available for testing. Thus, a total of 51 participants made up the case-cohort sample for this investigation (see Figure 1).

### Biomarkers

For the cohort reported herein, plasma stored at -80C was tested for C-reactive protein, cytokines, chemokines, and nutritional markers. These included the pro-inflammatory cytokines (TNFα, IL-6, INFγ, MIP-1α), anti-inflammatory cytokines (IL-10, IL-1ra), cytokines involved in innate and adaptive immunity (IL-15, IL-17, respectively), chemokines (MCP-1, MCP-3, IP-10), and nutritional markers (adiponectin, leptin). Levels were quantified using a Milliplex multi-analyte profiling assay (Millipore) in the University of Southern California Cancer Center Immunology Core Laboratory.

### Statistical analysis

Testing and estimation of the effect of each baseline biomarker on time to death were performed using Cox proportional hazards regression models adjusted for baseline CD4 cell count and HIV viral load. The biomarkers were log10 transformed and then modeled per 1 IQR increment in the Cox models. The Barlow weighting scheme was used to account for the case-cohort sampling. The Efron method was used to handle ties in failure times. Event times were calculated as the exact days from randomization date to the date of death.

Adjustments were not made for multiple comparisons. All analyses used SAS 9.4 with results considered significant when *P* < 0.05.

## RESULTS

At baseline, the case-cohort of 51 participants was comparable to the larger population of 282 participants in the parent cohort with CD4 cell count < 50 cells/mm^3^, with respect to age, sex, race, CD4 counts, and HIV viral load (*P* > 0.05 for all comparisons; data not shown). Of further importance, there was no difference in the proportion of participants randomized to earlier versus later ART.

For the cohort of 51 participants, the 33 who survived were not different from the 18 who died, in baseline characteristics that included age, sex, CD4 cell counts, HIV viral load, BMI, hemoglobin, albumin, adiponectin, leptin, or treatment assignment, except that the median age of the patients who died was 9 years older (*P* = 0.033; Table 1). Of note, 7 (14%) participants had BMI < 16.5 kg/m^2^ and 17 (33%) had BMI 16.5-18.5 kg/m^2^. The causes of death and week of death (in parentheses) in the ensuing year after randomization were disseminated tuberculosis (2, 4), gastroenteritis (3, 5, 11), pulmonary tuberculosis (4, 10), acute renal failure (5), bacterial pneumonia (8, 34), cryptococcal meningitis (9), bacterial meningitis (13), bacterial sepsis (14), peritonitis (11), intra-cranial hypertension (16), gunshot wounds (24), tuberculous meningitis (40), and no information (23). Death could not be related to treatment assignment (early versus deferred ART) in this small cohort.

**Table 1:** Baseline Clinical Characteristics and Markers of Nutrition

Median (Q1, Q3) or N (%)	Total (N = 51)	Deaths (N = 18)	Non-deaths (N = 33)	*P*-Value^a^
Age (years)	35 (30, 42)	42 (34, 45)	33 (30, 41)	0.033
Sex (male)	39 (76%)	13 (72%)	26 (79%)	0.732
CD4 count (cells/mm^3^)	25 (13, 36)	25 (8, 34)	25 (15, 38)	0.340
HIV RNA (log_10_copies/ml)	5.37 (5.00, 5.77)	5.40 (5.01, 5.78)	5.35 (5.00, 5.74)	0.771
Body Mass Index (BMI, kg/m^2^)	18.6 (17.3, 20.6)	18.3 (17.1, 20.3)	19.2 (17.6, 21.1)	0.955
< 16.5	7 (14%)	2 (11%)	5 (15%)	0.559
16.5 - 18.5	17 (33%)	8 (44%)	9 (27%)	
> 18.5	27 (53%)	8 (44%)	19 (58%)	
Hemoglobin (g/dl)	9.70 (8.40, 10.40)	9.45 (8.40, 10.10)	9.90 (8.40, 10.40)	0.484
Albumin (g/dl)	2.68 (2.38, 3.30)	2.61 (2.43, 3.00)	2.77 (2.30, 3.38)	0.365
Adiponectin (mg/dl)	3.50 (1.86,6.88)	3.48 (1.95, 5.00)	3.50 (1.86, 7.11)	0.400
Leptin (ng/ml)	0.20 (0.02, 0.85)	0.21 (0.02, 0.71)	0.06 (0.02, 1.14)	0.963
CD4/CD8 ratio^b^	0.07 (0.05, 0.14)	0.05 (0.02, 0.11)	0.07 (0.05, 0.14)	0.517
Treatment arm (immediate)	26 (51%)	9 (50%)	17 (52%)	>0.99

a) Fisher's exact test was used for analysis of sex, BMI group, and treatment arm; T-test with unequal variance was used for others;

b) Due to missing data, sample sizes were N = 32, N = 10, and N = 22, respectively.

For measures of nutrition, hazard ratios for average BMI, low BMI, serum hemoglobin, serum albumin, and leptin were not significant risk factors for death, although there was a trend for increased risk of death with advancing age (adjusted hazard ratio (HR) per 5 years increment = 1.32 [95% CI 0.97-1.79], *P* = 0.076, Table 2). Global inflammation measured by levels of C-reactive protein (CRP), pro-inflammatory cytokines (IL-6, TNFα, INFγ, IL-15, and IL-17) and chemokines MCP-3 and MIP was significantly higher in participants who died (Table 3). Increased risk of death was significantly associated with CRP (adjusted HR per 1 IQR width increment = 3.42 [95% CI 1.33-8.80], *P* = 0.011; Table 3), but the only pro-inflammatory cytokine associated with signifi-cant risk of death was IFNγ (adjusted HR = 2.46 [95% CI 1.02-5.90], *P* = 0.044). MCP-3 was associated with risk of death (adjusted HR = 3.67[95% CI 1.08-12.42], *P* = 0.037), but MCP-1 was not. Global measures of the innate (IL-15) and adaptive (IL-17) immune responses were both strongly associated with risk of death (adjusted HRs = 2.75 [95% CI 1.08-6.98], 3.99 [95% CI 1.06-15.07]; *P* = 0.033, 0.041, respectively).

**Table 2:** Risk of Death Associated with Age, Sex, and Baseline Markers of Nutrition

	Hazard Ratio (HR)^a^	95% Lower Confidence Limit for HR	95% Upper Confidence Limit for HR	*P*-Value Wald Test
Age (per 5 years increment)	1.32	0.97	1.79	0.076
Sex (male)	0.74	0.22	2.51	0.631
BMI (kg/m^2^)	1.07	0.81	1.42	0.646
BMI (ref: > 18.5) < 16.5	0.58	0.07	4.54	0.604
BMI (ref: > 18.5) 16.5 - 18.5	1.42	0.37	5.41	0.608
Hemoglobin (g/dl)	0.90	0.70	1.15	0.402
Albumin (g/dl)	0.78	0.38	1.60	0.493
Leptin^b^ (ng/dl)	1.29	0.52	3.20	0.582

a) Hazard ratios were adjusted for baseline CD4 cell count and HIV viral load;

b) More than 25% of measurements below level quantifiable.

**Table 3:** Markers of Inflammation at Baseline and Association with Risk of Death

Median (Q1, Q3)	Total (N = 51)	Deaths (N = 18)	Non-deaths (N = 33)	*P*-value T-test^a^	Hazard Ratio^b^ (95% CI) per 1 IQR increment	*P*-Value Wald Test
CRP (mg/dl)	9.88 (3.46, 28.3)	15.4 (8.47, 50.8)	7.19 (2.43, 18.2)	0.004	3.42 (1.33, 8.80)	0.011
IL-1RA^c^ (ng/ml)	22.3 (2.9, 88.4)	41.5 (13.2, 88.4)	2.9 (2.9, 82.2)	0.076	1.96 (0.81, 4.73)	0.135
IL-6 (ng/ml)	6.1 (1.3, 16.5)	9.8 (2.8, 20.1)	3.9 (0.8, 12.2)	0.040	2.58 (0.79, 8.38)	0.115
IL-8 (ng/ml)	11.5 (6.5, 21.8)	17.6 (11.5, 23.9)	9.5 (5.9, 17.6)	0.024	1.94 (0.99, 3.80)	0.054
IL-10 (ng/ml)	16.9 (3.7, 41.5)	37.8 (8.0, 52.5)	11.5 (3.7, 23.6)	0.501	1.15 (0.55, 2.43)	0.705
IL-15^d^ (ng/ml)	0.4 (0.4, 7.5)	7.0 (0.4, 8.6)	0.4 (0.4, 5.7)	0.029	2.75 (1.08, 6.98)	0.033
IL-17 (ng/ml)	3.2 (0.2, 13.1)	9.7 (1.7, 14.9)	1.4 (0.2, 6.6)	0.025	3.99 (1.06, 15.07)	0.041
IP-10 (ng/ml)	2.89 (1.78, 4.72)	3.18 (1.75, 5.02)	2.51 (1.90, 4.46)	0.848	1.23 (0.54, 2.82)	0.625
MCP-1 (ng/ml)	300 (224, 438)	377 (224, 590)	299 (229, 349)	0.256	1.89 (0.78, 4.60)	0.160
MCP-3^e^ (ng/ml)	20.6 (2.0, 36.1)	25.5 (16.1, 52.7)	11.1 (2.0, 33.5)	0.027	3.67 (1.08, 12.42)	0.037
MIP (ng/ml)	34.9 (20.7, 49.1)	41.9 (30.0, 49.2)	28.4 (14.1, 47.8)	0.028	1.76 (0.95, 3.26)	0.073
TNF (ng/ml)	27.5 (17.0, 35.6)	34.0 (27.5, 47.6)	24.0 (16.0, 30.6)	0.022	2.08 (0.91, 4.77)	0.083
IFNγ (ng/ml)	22.5 (9.6, 38.7)	25.2 (15.2, 54.4)	21.5 (8.2, 35.7)	0.038	2.46 (1.02, 5.90)	0.044
Adiponectin (mg/dl)	3.50 (1.86, 6.88)	3.48 (1.95, 5.00)	3.50 (1.86, 7.11)	0.400	0.83 (0.46, 1.49)	0.528

a) Log10 transformed data were used for statistical analysis;

b) Hazard Ratios were adjusted for baseline CD4 cell count and HIV viral load;

c, d, e) More than 25% of measurements below level quantifiable.

## DISCUSSION

Results of our investigation showed that body mass and some markers of nutrition (albumin, leptin, and hemoglobin) were not related to an increased risk of death in dually-infected participants after initiating anti-tuberculous therapy and ART. Whereas, inflammation as assessed by C-reactive proteins (CRP), pro-inflammatory cytokines, chemokines, and biomarkers of the innate and adaptive immune response were all significantly higher in participants who died than in those who survived for the ensuing year.

The lack of association of nutritional markers in cases of death was somewhat surprising since low BMI, presumably related to inadequate energy intake and incident catabolism/cachexia is not only a risk factor for tuberculosis but also for death, especially in sub-Saharan Africa. In our cohort and the parent study, many of participants were from Sub-Saharan Africa [3]: approximately 50% had a BMI <18.5kg/m^2^ and approximately15% had a BMI < 16.5 kg/m^2^, indicating that they were quite malnourished. Although good nutritional support during early treatment has sound underpinnings [30, 31], our findings do not support the idea that nutritional supplementation during early treatment will favorably affect survival in the following 12 months for treatment-naive participants with HIV and dually-infected with both pathogens.

The hazard ratio for CRP, a global measure of inflammation, was more than 3-fold higher in participants dying than those surviving. Although understanding the specific immune response to clinical tuberculosis is complex, it appears to begin with an innate immune response to tuberculous antigens [32]. This immediate response is mediated by natural killer (NK) lymphocytes that are cytotoxic and lyse autologous infected cells [33–35]. A small fraction of NK cells also secrete IFNγ to activate monocytes and macrophages [33, 34, 36, 37] to kill intracellular cellular organisms [38–41], both necessary to control bacillary replication. The NK cells upregulate CD8+IFNγ+ lymphocytes to stimulate infected monocytes (presumably macrophages as well) to secrete the pro-inflammatory cytokine, IL-15 [42], which is a cardinal marker of the innate immune response, and IL-15 is also upregulated by TLR1/2 signaling during mycobacterial infection to trigger macrophage differentiation [43]. Levels of both IL-15 and INFγ were significantly elevated in our study participants who died compared to those who survived.

The adaptive immune response is orchestrated largely by T lymphocytes in response to *M. tuberculosis* [44], which generate cytotoxicity by activated CD8 cells and cytokine secretion [40, 41]; the 2 primary pro-inflammatory cytokines released are INFγ and IL-17 [45, 46]. Indeed, most of the IFNγ generated during tuberculosis infection is secreted by T cells and is necessary for the microbicidal function of macrophages to eradicate *M. tuberculosis* [40, 41, 47]. IFNγ upregulates IL-17, which is generated by Th-17 cells [48] and among other actions recruits neutrophils to the site of infection [49], mediates macrophage accumulation [50], and feeds back to induce Th-1 cells to secrete more IFNγ [51]. In our cohort, levels of IL-17, a biomarker of the adaptive immune response, were also increased to a greater degree in participants dying in our study.

The timing and interactions of the innate and adaptive immune systems are complex. Data suggest that NK cells (innate immune response) link these 2 systems by optimizing CD8 lymphocytes (adaptive immune response) to secrete IFNγ needed to lyse *M. tuberculosis*-infected cells [32]. Other data suggest the γδ T cells (adaptive response), which are preferentially expanded in HIV-infected patients and which secrete pro-inflammatory cytokines including IFNγ and IL-17 [51, 52], coordinate and bridge the innate and adaptive immune response [53–57]. Furthermore, IL-15 secreted by phagocytic monocytes during the innate response is a growth factor for γδ T cells, promotes cytotoxicity by NK cells, and stimulates their secretion of INFγ and TNFα [58], thereby linking innate and adaptive immunity during infection with *M. tuberculosis* [59].

Co-infection with HIV contributes to the complexity of the clinical outcomes and immune response in dual infection with HIV and *M. tuberculosis* [60, 61]. Patients infected with HIV have a greater risk of developing active tuberculosis after exposure *to M. tuberculosis* and extra-pulmonary disease is associated with more severe HIV immunodeficiency [13]. Conversely, tuberculosis can accelerate HIV infection [62, 63], and HIV-infected patients co-infected with tuberculosis have shorter survival compared to age- and CD4-matched patients who are HIV-positive but not infected with *M. tuberculosis* [62, 64]. In particular, tuberculosis increases HIV viral replication 5-160 fold, primarily in activated T cells [65]. Furthermore, HIV and tuberculous co-infection is associated with higher mortality in the setting of low CD4 cell counts [15–17]. Although all participants selected for our case-cohort had low CD4 counts, we still adjusted our baseline measures for CD4 cell counts and HIV viral load.

In addition, it has recently been demonstrated that mortality in HIV and *M. tuberculosis* co-infected patients is linked to immune dysfunction of monocytes, especially related to inflammatory mediator production of IL-6, TNFα, and CSF3 and expansion of CD16+CD14+ monocytes [66]. The mechanism that drives this inflammatory response may be through INFγ stimulated indolamine 2, 3 dioxygenase (IDO) production by monocytes [67]. In fact, it was recently shown that IDO was a very strong predictive biomarker of active tuberculosis in HIV co-infected patients [68]. We found a significant association between INFγ and death in our cohort. Moreover, the kynurenine pathway is an important regulator of both the innate and adaptive immune response [69], which were upregulated in our death cohort. Thus, further studies in our cohort evaluating IDO activity and the levels of kynurenine that results from the breakdown of tryptophan by IDO are warranted.

Our study has several limitations. The case-cohort for whom stored samples were available was relatively small, which limited the power to detect other associations between biomarkers and risk of death. The availability of stored plasma may have potentially introduced bias due to possible differences between participants with and without stored plasma. In addition, the cross-sectional nature does not allow causality to be established, but only generates conceptual linkages by inference for hypothesis testing in future, larger prospective studies. We used multiplex cytokine methodology, whereas individual platform assays may have performed differently for some of the biomarkers, such as for the discrepancy between MCP-1 (not elevated) and MCP-3 (elevated) in cases of death. Yet, there was internal consistency for the immune response to *M. tuberculosis* as evidenced by significant increases in IFNγ, IL-15, and IL-17 in cases of death, a profile typical of *M. tuberculosis* mono-infected patients [32, 53]. Indeed, activation of both the innate and adaptive immune responses was exaggerated in our dying participants. Finally, because PBMCs collected from the case-cohort were not viable, we could not measure cellular activation markers.

Regardless, treatment naive participants dying in the months after enrolling in the study had more profound inflammation, which appeared to be globally associated with activation of both innate and adaptive immunity. Future studies that include quantification of different cellular phenotypes and their activation in addition to soluble markers will be needed to unravel the mechanisms underlying our important preliminary observations related to inflammation-associated risk for death in these dually infected patients.
